# Molecular docking, 3D-QSAR and structural optimization on imidazo-pyridine derivatives dually targeting AT1 and PPARγ

**DOI:** 10.18632/oncotarget.15778

**Published:** 2017-02-28

**Authors:** Jun Zhang, Qing-Qing Hao, Xin Liu, Zhi Jing, Wen-Qing Jia, Shu-Qing Wang, Wei-Ren Xu, Xian-Chao Cheng, Run-Ling Wang

**Affiliations:** ^1^ Tianjin Key Laboratory on Technologies Enabling Development of Clinical Therapeutics and Diagnostics (Theranostics), School of Pharmacy, Tianjin Medical University, Tianjin 300070, China; ^2^ Tianjin Key Laboratory of Molecular Design and Drug Discovery, Tianjin Institute of Pharmaceutical Research, Tianjin 300193, China

**Keywords:** imidazo-\pyridines, AT1, PPARγ, molecular docking, 3D-QSAR

## Abstract

Telmisartan, a bifunctional agent of blood pressure lowering and glycemia reduction, was previously reported to antagonize angiotensin II type 1 (AT1) receptor and partially activate peroxisome proliferator-activated receptor γ (PPARγ) simultaneously. Through the modification to telmisartan, researchers designed and obtained imidazo-\pyridine derivatives with the IC_50_s of 0.49∼94.1 nM against AT1 and EC_50_s of 20∼3640 nM towards PPARγ partial activation. For minutely inquiring the interaction modes with the relevant receptor and analyzing the structure-activity relationships, molecular docking and 3D-QSAR (Quantitative structure-activity relationships) analysis of these imidazo-\pyridines on dual targets were conducted in this work. Docking approaches of these derivatives with both receptors provided explicit interaction behaviors and excellent matching degree with the binding pockets. The best CoMFA (Comparative Molecular Field Analysis) models exhibited predictive results of q^2^=0.553, r^2^=0.954, SEE=0.127, r^2^_pred_=0.779 for AT1 and q^2^=0.503, r^2^=1.00, SEE=0.019, r^2^_pred_=0.604 for PPARγ, respectively. The contour maps from the optimal model showed detailed information of structural features (steric and electrostatic fields) towards the biological activity. Combining the bioisosterism with the valuable information from above studies, we designed six molecules with better predicted activities towards AT1 and PPARγ partial activation. Overall, these results could be useful for designing potential dual AT1 antagonists and partial PPARγ agonists.

## INTRODUCTION

Type 2 diabetes mellitus (T2DM) was thought to result from the combination of genetic factor, such as lifestyle changing, population aging, exercise reducing [1, 2]. It is predicted that the number of people with this disorder will be increasing without highly efficient therapy [3, 4]. In addition, diabetes mellitus was thought to exhibit some relationship with hypertension. The incidence of hypertension occurring to patients with diabetes mellitus is almost twice higher than those with no diabetes mellitus. The greater mortality of patients in diabetes mellitus right attributed to cardiovascular diseases, among which hypertension takes up to high proportion of 75% [5]. So, developing novel and potent agents to concurrently treat hyperglycemia and hypertension with high occurrence allows of no delay.

Telmisartan, which antagonized angiotensin II type-1 receptor (AT1R), was a novel oral agent for blood pressure reducing and cardiovascular protection [6]. Previously, it was reported to display dual activities of antagonizing AT1 and partially activation towards PPARγ [7–9].

The peroxisome proliferator-activated receptors (α, δ and γ) belong to the nuclear hormone receptor superfamily that regulate the expression of target genes [10, 11]. PPARγ, the most thoroughly studied isoform in the treatment of metabolic syndrome [12], is widely expressed in adipose tissue, macrophages, liver, kidney and lung [13]. The binding of active ligands to PPARγ would modulate the expression of related genes, play vital role in lipogenesis, glycolipids metabolism and immune system [14]. Thiazolidinediones (TZDs, such as rosiglitazone and piglitazone) can greatly activate PPARγ. TZDs were first reported as insulin-sensitizing drugs in the early 1980s by the pharmaceutical company Takeda and developed for the treatment of type 2 diabetes mellitus in clinical practice [15–17]. However, the administration of TZDs could produce severe side effects such as fluid retention, weight gain, cardiac hypertrophy, bone fractures, and hepatotoxicity [18]. As reported in 2007 by Nissen and Wolski, rosiglitazone was removed from the European market due to its association with excessive cardiovascular risk [12, 19, 20].

Renin-angiotensin system (RAS) has reported to exhibit significant roles in reducing blood pressure and maintaining electrolyte and fluid homeostasis. Angiotensin receptor included in this system is a hypertension-related G protein-coupled receptor (GPCR) [21]. There are two main types: AT1 and angiotensin II type-2 receptor (AT2), among which the former receives the most research [22]. The bio-effects of angiotensin II (Ang II), such as vasoconstriction, the increase of vasopressin secretion and myocardial hypertrophy, are primarily developed via the activation of AT1 while activating AT2 will inhibit cell growth and lead to cell differentiation and apoptosis [23–25]. AT1 receptor blockers (ARBs), namely sartans, which enable to block the action of AT1 receptor, were developed to treat unfavorable symptoms [9, 26, 27].

**Figure 1:**
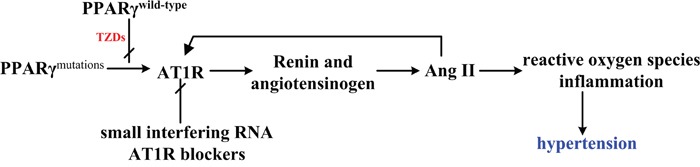
The closely relationship between PPARγ and RAS (rennin-angiotensin system) As shown, PPARγ mutations would increase the expression of AT1 receptor, thus leading to hypertension through produced reactive oxygen species (ROS) and inflammation. However, treatment of TZDs, small interfering RNA and AT1R blockers will interrupt this effect by interfering different phases.

As summarized in Figure 1, PPARγ closely correlated with RAS (rennin-angiotensin system). The mutation of PPARγ would induce the enhanced expression of AT1R, leading to an increase of Ang II. As a result, the following reactive oxygen species cause hypertension symptom. Fortunately, the reported PPARγ agonists like TZDs could activate PPARγ as well as block its mutation, thus interrupting the following process just as the role of small interfering RNA and AT1R blockers [28].

**Figure 2:**
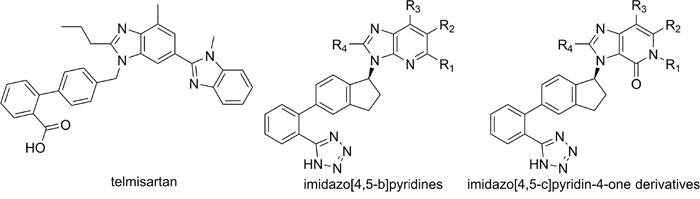
Structural scaffolds of imidazo[4,5-b]pyridines and imidazo[4,5-c]pyridin-4-one derivatives modified from telmisartan

In order to find novel drugs with dual AT1 antagonism and partial PPARγ activation activities, the structure of telmisartan (Figure 2) could be modified to retain the AT1 receptor antagonistic activity and enhance the partially PPARγ agonistic activity. Series of imidazo[4,5-b]pyridines and imidazo[4,5-c]pyridin-4-one derivatives (Figure 2) were obtained by Agustin Casimiro-Garcia [17, 29] via maintaining the main scaffold of telmisartan. These compounds showed robust AT_1_ antagonistic activity and partial activation of PPARγ. Their activities were evaluated with the IC_50_s of 0.49∼94.1 nM against AT1 and EC_50_s of 20∼3640 nM for PPARγ partial activation.

For the purpose of minutely determining the binding mode and structure-activity relationship (SAR) of these molecules, docking and CoMFA (Comparative Molecular Field Analysis) studies were conducted in our work. Wholly, these results from the computational techniques could be useful for designing potential dual AT1 antagonists and PPARγ partial agonists.

## RESULTS AND DISCUSSION

### Molecular docking

**Figure 3:**
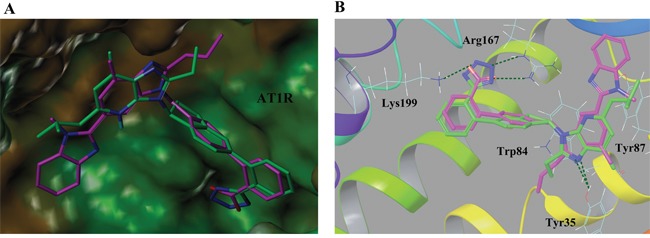
(A) Binding surface of compound 8 (green) and telmisartan (magenta) with AT1 receptor pocket (PDB ID: 4ZUD) **(B)** Interaction behaviors of compound 8 (green) and telmisartan (magenta) in AT1 receptor active site. H-bond interactions were represented by green dashed line. Telmisartan was regarded as a positive control.

For a deep insight into the crucial interactions of molecules with AT1 and PPARγ, these imidazo-\ pyridine derivatives (Table 1) were docked into the protein's binding site using standard-precision (SP) docking tool [30]. As presented in Figure 3, the fascinating illustrations of ligands (8 and telmisartan) with AT1 pocket could be of great help to understand the binding mechanism. The whole structural skeletons of both molecules were appropriate for the pocket environment. The representative compound 8 superimposed perfectly with telmisartan in the binding surface, especially the hydrophobic moiety as well as the parts of hydrogen donors and acceptors, which validated the similar modes to telmisartan. Additionally, telmisartan matched better with the receptor surface compared with compound 8, illustrating the higher AT1 antagonistic activity than 8. This was mainly derived from the different R substituent in structures, which led to some divergence in position or orientation in the binding surface. As to the detailed interactions in AT1 pocket, the critical hydrogen bonds of compound 8 with residues Tyr35, Arg167 and Lys199 were almost consistent with telmisartan. Both molecules formed π-π interaction with Trp84 as well. Above-mentioned results from docking analysis verified the bio-activities of these compounds against AT1 receptor.

**Table 1:** Structures and bioactivities (AT1-IC_50_ and PPARγ-EC_50_) of imidazo-\ pyridine derivatives from available literatures [17, 29] 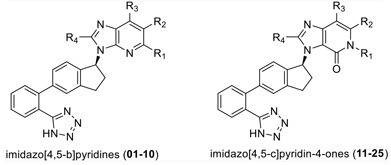

No.	R_1_	R_2_	R_3_	R_4_	AT1 IC_50_(nM)	PPARγ EC_50_(nM)
**Telmisartan**					0.49	1520
**1**	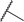	H	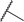	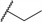	7.6	591
**2**	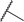	H	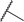	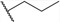	13.3	1320
**3**	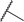	H	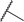	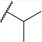	10.2	295
**4**	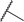	H	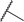	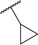	8.2	762
**5**	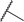	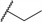	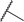	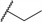	15.7	1340
**6**	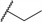	H	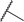	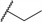	6.8	494
**7**	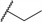	H	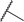	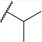	16.9	264
**8**	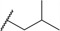	H	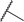	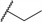	1.6	212
**9**	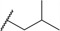	H	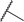	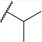	3.5	89
**10**	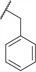	H	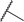	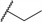	5.2	90
**11**	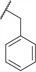	H	H	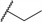	12.7	292
**12**	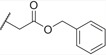	H	H	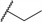	19.8	1250
**13**	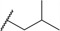	H	H	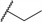	19.4	1710
**14**	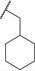	H	H	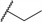	41	942
**15**	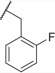	H	H	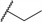	37.7	103
**16**	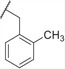	H	H	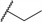	5.1	97
**17**	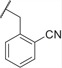	H	H	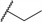	13.5	685
**18**	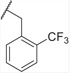	H	H	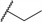	94.1	187
**19**	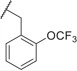	H	H	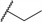	29.3	20
**20**	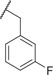	H	H	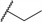	67.4	159
**21**	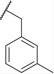	H	H	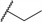	36.9	108
**22**	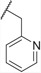	H	H	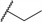	63.8	3580
**23**	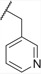	H	H	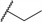	45.2	3640
**24**	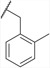	H	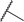	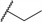	6.8	42
**25**	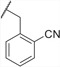	H	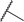	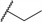	7	295

**Figure 4:**
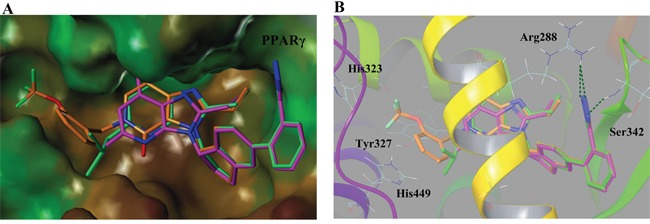
(A) Binding surface of compound 8 (green), 19 (orange) and co-crystallized ligand 1 (magenta) with PPARγ pocket (PDB ID: 3R8A) **(B)** Interaction behaviors of compound 8 (green), 19 (orange) and co-crystallized ligand 1 (magenta) in PPARγ active site. H-bond interactions were represented by green dashed line. Co-crystallized ligand 1 was regarded as a positive control.

Seen from Figure 4A, compound 8, 19 and co-crystallized ligand 1 matched well in PPARγ binding surface. As R_1_ substituent was radically diverse, the orientation of this part in the surface was somewhat inconsistent with each other. The R_1_ part of compound 19 obviously extended to the narrow pocket, so compound 19 was in perfect accordance with PPARγ pocket in comparison to 1 and 8, validating its higher agonistic activity. Therefore, a proper modification to R_1_ will probably be beneficial to the PPARγ partial activity, such as increasing the substituent, extending the carbon chain.

Unexpectedly in PPARγ interaction modes, the lipophilic tails (R_1_) of 1, 8 and compound 19 were buried in AF-2 domain of the binding pocket, interacting with residues His323, Tyr327 and His449 through non-polar or van der Waals interactions as indicated in previous paper [17, 29]. Clearly, their interaction modes were substantially distinct from the typical PPARγ agonists, whose acid heads directly interacted with His323, Tyr327 and His449. Although the acidic tetrazole ring of 1, 8 and 19 bound well in similar orientation, their binding behavior in the active site displayed some different. The N-2 of tetrazole ring in molecule 1 and 8 interacted with Arg288 through forming an H-bond while 19 formed another H-bond between the N-1 of tetrazole and NH of Ser342, which was mainly due to the structural flexibility.

### CoMFA statistical analysis

#### AT1 model

The CoMFA model was derived using a training set of 26 imidazo-\ pyridines including 10 imidazo[4,5-b]pyridines and 15 imidazo[4,5-c]pyridin-4-one derivatives with telmisartan as the template. The test set including five molecules was to validate the external predictive power of the CoMFA model. The predictive and residual values of the dataset were mentioned in Table 2, and other statistical results from the best model were listed in Table 3.

**Table 2:** The actual and predictive activity values of the training and test datasets from the best AT1 and PPARγ CoMFA models

No.	AT1			PPARγ		
	Actual pIC_50_	Predicted pIC_50_	Residual error	Actual pEC_50_	Predicted pEC_50_	Residual error
**Telmisartan**	0.3098	0.222	0.0879	-3.1818	-3.181	-0.0008
**1**	-0.8808	-1.008	0.1271	-2.7716	-2.781	0.0096
**2**	-1.1239	-1.035	-0.0888	-3.1206	-3.109	-0.0114
**3**	-1.0086	-1.051	0.0429	-2.4698	-2.473	0.0032
4^#^*	-0.9138	-0.846	-0.0678	-2.8820	-3.379	0.4967
**5**	-1.1959	-1.117	-0.079	-3.1271	-3.121	-0.0063
**6**	-0.8325	-0.873	0.0406	-2.6937	-2.719	0.0252
**7**	-1.2279	-0.923	-0.3054	-2.4216	-2.404	-0.0172
8*	-0.2041	-0.418	0.2141	-2.3263	-2.326	-0.0003
**9**	-0.5441	-0.496	-0.0482	-1.9494	-1.954	0.0049
10*	-0.7160	-0.636	-0.0804	-1.9542	-2.572	0.6178
**11^#^**	-1.1038	-1.618	0.5142	-2.4654	-2.431	-0.034
12*	-1.2967	-1.262	-0.0344	-3.0969	-2.569	-0.5279
**13**	-1.2878	-1.363	0.0756	-3.2330	-3.215	-0.0179
**14**	-1.6128	-1.53	-0.0829	-2.9741	-2.985	0.0106
**15**	-1.5763	-1.455	-0.1217	-2.0128	-2.014	0.0013
**16^#^**	-0.7076	-1.41	0.7024	-1.9868	-2.003	0.0166
**17**	-1.1303	-1.028	-0.1021	-2.8357	-2.842	0.0061
**18**	-1.9736	-2.091	0.1173	-2.2718	-2.27	-0.0016
**19**	-1.4669	-1.466	-0.0011	-1.3010	-1.304	0.0035
20^#^*	-1.8287	-1.717	-0.1117	-2.2014	-2.736	0.5346
**21**	-1.5670	-1.675	0.1075	-2.0334	-2.046	0.0128
**22**	-1.8048	-1.762	-0.0427	-3.5539	-3.564	0.0102
**23**	-1.6551	-1.773	0.1175	-3.5611	-3.565	0.0036
**24^#^**	-0.8325	-1.325	0.4925	-1.6232	-1.611	-0.0125
**25**	-0.8451	-0.901	0.0561	-2.4698	-2.464	-0.0057

# test set of AT1 model, * test set of PPARγ model.

**Table 3:** Statistical parameters from the best AT1 and PPARγ CoMFA models

Statistical Parameters		CoMFA	
		AT1	PPARγ
Leave-One-Out(LOO)	q^2^	0.553	0.503
	ONC	3	10
	r^2^	0.954	1.000
No validation	SEE	0.127	0.019
	F value	117.807	2107.933
	r_pred_^2^	0.779	0.604
Field distributions (%)	Steric	61.9	75.4
	Electrostatic	38.1	24.6

a: the cross-validated correlation coefficient, q^2^; b: the optimum number of components, ONC; c: the conventional correlation coefficient, r^2^: d: standard error of estimate, SEE; e: Fisher Test value; f: the predicted correlation coefficient, r^2^_pred_.

**Figure 5:**
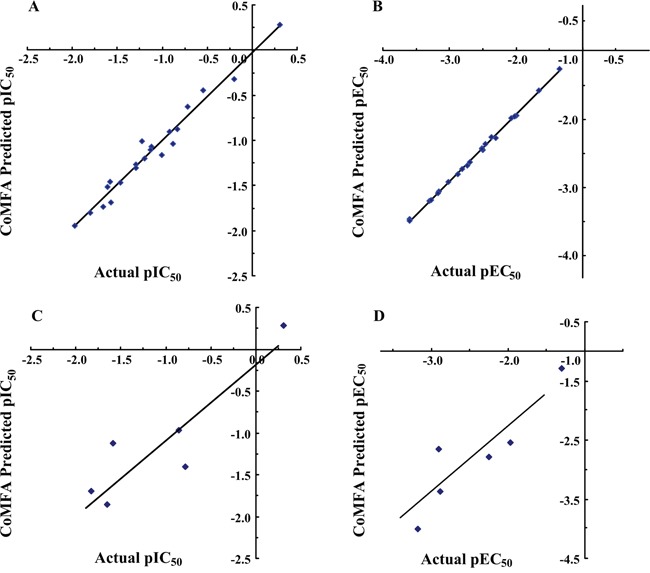
The graphical presentations of the predicted activities versus the actual activities using the best CoMFA models **(A)** Fitted prediction for the training set of best AT1 model (r^2^=0.954). **(B)** Fitted prediction for the training set of best PPARγ model (r^2^=1.00). **(C)** Fitted prediction for the test set of best AT1 model (r^2^_pred_=0.779). **(D)** Fitted prediction for the test set of best PPARγ model (r^2^_pred_=0.604).

As shown in Table 3, the cross-validated correlation coefficient q^2^ through LOO method was 0.553 and the number of optimal components was 3. With the observed optimal ONC, the favorable conventional correlation coefficient r^2^ (0.954), SEE (0.127) and F value (117.807) from no validation method implied a highly qualified and robust CoMFA model. Figure 5 illustrated an excellent agreement between the actual pIC_50_s and the predicted values of the training set (Figure 5A) and the test set (Figure 5C). Nevertheless, the predicted correlation coefficient r^2^_pred_ (0.779) from the test set confirmed the CoMFA model to be highly predictive. The distributions for steric and electrostatic fields were 61.9% and 38.1%, respectively, suggesting a higher steric contribution to AT1 antagonistic activity than electrostatic field.

#### PPARγ model

A training set of 21 imidazo-\ pyridines and a test set of five other derivatives were employed to construct and validate the CoMFA model, respectively. The statistical data were summarized in Table 2 and Table 3. PLS method yielded the terms of q^2^ (0.503) with ten principal components, r^2^ (1.00), SEE (0.019) and F value (2107.933). Similarly to AT1, steric field (75.4%) was found to exhibit an absolutely higher contribution towards PPARγ partial agonism compared to the electrostatic field (24.6%). The linear regression curve portrayed in Figure 5D (r^2^_pred_=0.604) along with the q^2^ value (>0.6) implied the CoMFA model to be relatively robust and stable.

The satisfactory statistical results demonstrated the predictivity and accuracy of the CoMFA models (AT1 and PPARγ). The qualified models could be used in further study to evaluate and design novel imidazo-\ pyridine derivatives with potential AT1 antagonism and PPARγ partial activation.

### The StDev*Coefficient CoMFA contour maps

**Figure 6:**
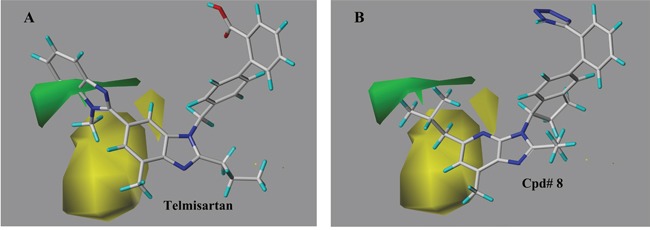
The steric field contour maps of representative compounds from the best CoMFA model (AT1) Green regions referred to higher activity by introducing bulker groups while the yellow parts would lead to increased activity with smaller substituents.**(A)** telmisartan (the most active molecule), **(B)** compound 8.

**Figure 7:**
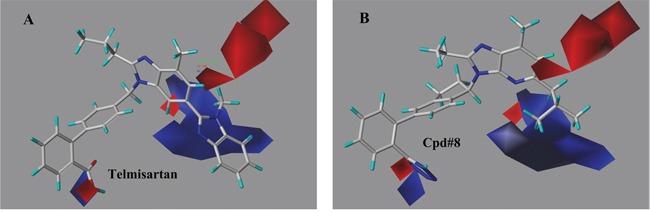
The electrostatic field contour maps of representative compounds from the best CoMFA model (AT1) The blue and red contours respectively indicated that electropositive and electronegative substituents in the corresponding position would lead to increased antagonistic activity. **(A)** telmisartan (the most active molecule), **(B)** compound 8.

**Figure 8:**
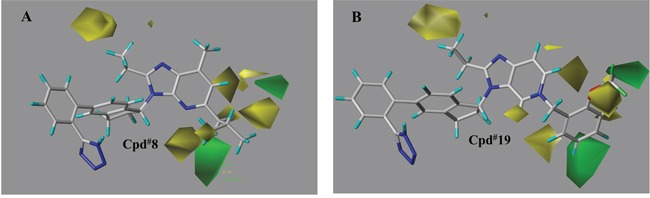
The steric field contour maps of representative compounds from the best CoMFA model (PPARγ) The sterical favourable and unfavourable regions were represented in green and yellow, respectively. **(A)** compound 8, **(B)** compound 19 (the most active molecule).

**Figure 9:**
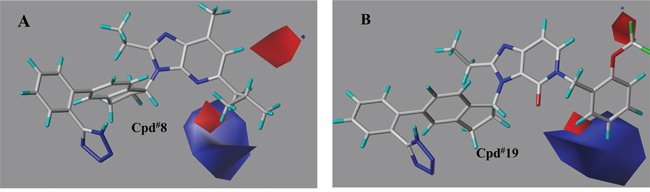
The electrostatic field contour maps of representative compounds from the best CoMFA model (PPARγ) The blue and red contours respectively indicated that electropositive and electronegative substituents in the corresponding positions would lead to increased inhibitory activity. **(A)** compound 8, **(B)** compound 19 (the most active molecule).

The contour plots of the steric and electrostatic fields observed from the best CoMFA models of both targets were depicted in Figures 6–9. The type of CoMFA field was regulated to StDev*Coeff (the standard deviation and the coefficient). The field levels for favored and disfavored regions were set to be default (80.0% and 20.0%, respectively). In steric field contour maps (Figure 6 and Figure 8), green blocks referred to areas where increasing the steric bulk of one substituent would enhance the activity while yellow areas represented the opposite. Similarly in electrostatic field maps (Figure 7 and Figure 9), blue contours indicated a higher bioactivity by introducing electron-donating groups. The red parts, however, presented that the potency will be improved with the electron withdrawing substituent.

#### AT1 model

As interpreted in Figure 6, there was a large yellow polyhedron falling between the benzyl of the central benzimidazole ring and the imidazole group of the distal benzimidazole, indicating a greater influence on AT1 antagonistic activity. Accordingly, properly reducing the volume of the substituent would favor a lot to the activity. A green polyhedron with small size mapping the benzyl of the distal benzimidazole was hypothesized that a little increase to the bulk of the substituent on the benzyl would be beneficial. According to the CoMFA map of compound 8, a similar yellow piece overlapped the half of pyridine in the imidazo[4,5-b]pyridine, which suggested that the volumes of particular R_2_ part were negatively correlated to AT1 antagonistic activity. Therefore, compound 5 (15.7nM) holding an ethyl group in R_2_ position reduced the role in antagonism compared to compound 1 (7.6nM) with no substitution. Absolutely greater potency to 8 (1.6nM) and 9 (3.5nM) over compound 1 (7.6nM), 6 (6.8nM) and 3 (10.2nM) respectively just exemplified the significant role by the green block near R_1_ part.

The contributions of the electrostatic field to the representative telmisartan and 8 were described in Figure 7. In details for the contour map of telmisartan, a large blue contour mapping the imidazole group of the distal benzimidazole indicated that the electropositive substituent was preferred for greater effect against AT1 activity. The red parts located in the hydroxyl of carboxyl group and the C-4 position of the central benzimidazole referred that the electronegativity of the groups at this position tended to be beneficial to the activity. Taking compound 8 for further analysis, there was a big blue piece located around R_1_, perfectly illustrated a better activity of compound 8, 9 with isobutyl and 10 with benzyl than compounds with methyl in R_1_ part. Besides, compound 16 with an methyl group substituting R_1_ part showed clearly higher potency than compound 15, 17, 18, 19 and 20 with electron withdrawing -F, -CN, -CF_3_,-OCF_3_ and -F group. Several red blocks around R_2_ moiety indicated a potentially better efficacy if with electron withdrawing groups.

#### PPARγ model

The contributions of steric and electrostatic fields to PPARγ partial activation were 75.4% and 24.6%, respectively. So the sterical bulk played decisive role in controlling the PPARγ partial agonism. Figure 8 illustrated the steric contour maps of 8 and 19 towards PPARγ model. Seen from Figure 8A, a large green region located near R_1_, illustrating a better PPARγ activity if with properly bigger substituent, which could be verified by compound 1 (591nM), 6 (494nM), 8 (212nM) and 10 (90 nM). Around part R_2_, there were several small yellow blocks, indicating that groups with small volume were beneficial to the partial agonism activity. That was why –H was applied to R_2_ of imidazo[4,5-b]pyridines and imidazo[4,5-c]pyridin-4-one derivatives. Additionally, a single big yellow piece situated near R_4_ moiety, so molecule 2 (1320nM) with n-propyl tended to be less active than 1 (591nM) with anethyl group. As observed from the study, ethyl tended to be an excellent group for potent PPARγ partial activation. As to the series of imidazo[4,5-c]pyridin-4-one derivatives (Figure 8B), a small green block near -OCF_3_ of part R_1_ (19) exactly explicated the higher potency than 18 with -CF_3_ group and 15 with -F substituted. An independent green piece mapped the vicinity between C-4 and C-5 position in molecule 19, assuming that the bulker group in this part would lead to increased agonistic activity.

Seen from Figure 9A, a large blue block as well as a small red piece lay around part R_1_ of compound 8, supposing an increase in PPARγ partial activity with electron-donating groups. Additionally in Figure 9B, a relatively small red region stretched into C-2 position of part R_1_, validating that molecule 11 (292nM) with –H, 15 (103nM) with -F atom, 16 (97nM) with –CH_3_, 17 (685nM) with -CN, 18 (187nM) with –CF_3_ group displayed lower activity than molecule 19 (20nM). The larger blue block in C-5 position of part R_1_ assumed that the PPARγ partial activation may enhance if substituted with electron-donating groups. Additionally, the small red region around 6 position of R_1_ (Figure 9B) might induce increased agonism by electron-withdrawing groups.

**Figure 10:**
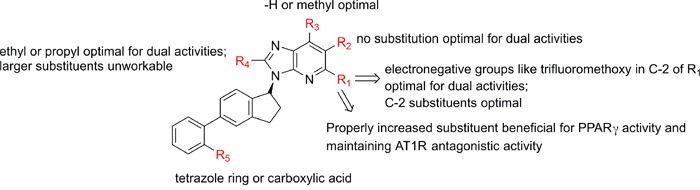
The SAR of imidazo-\ pyridine derivatives from the best CoMFA models (AT1 and PPARγ)

With overall analysis to imidazo-\ pyridine derivatives, we could summarize the rules (Figure 10) as follows: (1) Increasing the R_1_ substituent properly will be beneficial to enhance PPARγ partial activity and maintain AT1R antagonistic activity; (2) The electronagative groups like trifluoromethoxy in C-2 of part R_1_ caused the dual activities to increase and compounds with 2-substituted groups tended to be more active than that of other positions; (3) R_2_ substitution was improper for enhancing the activities towards AT1R antagonism and PPARγ partial activation; (4) ethyl or propyl in R_4_ was appropriate for dual activities, larger substituents were unworkable; (5) Tetrazole ring or carboxylic acid in R_5_ was responsible for better dual activities.

### Structural optimization

Considering the structural factors towards AT1 and PPARγ partial activities, six molecules (Table 4) were rationally modified and screened based on the structure of compound 8. In the design process, we replaced the tetrazole ring with carboxylic acid group via the bioisostere principle and primarily substituted R_1_ and R_3_ parts with proper groups according to the contour maps from best AT1/PPARγ CoMFA models. The activities of these designed structures towards AT1 and PPARγ were predicted to be almost better compared to that of reported imidazo[4,5-b]pyridines and imidazo[4,5-c]pyridin-4-one derivatives. The successful molecule design above illustrated that the constructed CoMFA models were highly stable and practicable to acquire novel, potential dual AT1 antagnists and PPARγ partial agonists.

**Table 4:** The structures and predicted AT1/PPARγ activities of new designed molecules based on the best CoMFA models 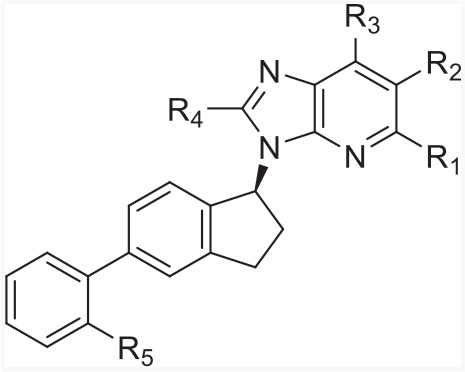

No.	R_1_	R_2_	R_3_	R_4_	R_5_	pIC_50_(AT1)	pEC_50_(PPARγ)
**A1**	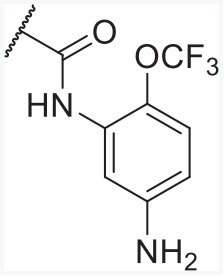	H	H	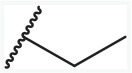	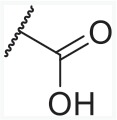	-0.429	-1.735
**A2**	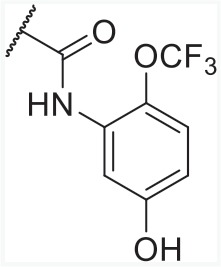	H	H	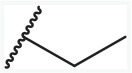	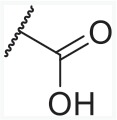	-0.479	-1.767
**A3**	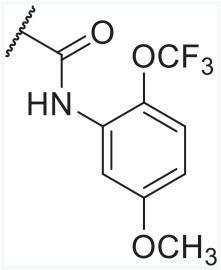	H	H	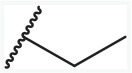	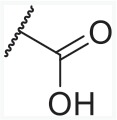	-0.497	-1.763
**A4**	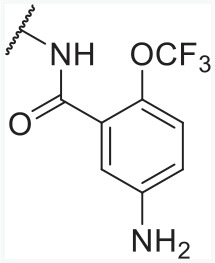	H	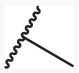	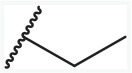	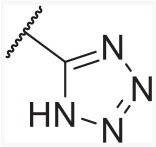	-0.654	-1.512
**A5**	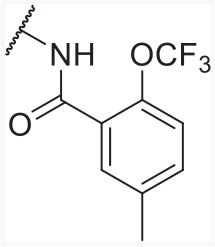	H	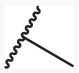	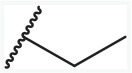	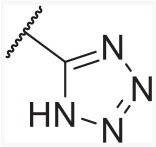	-0.657	-1.544
**A6**	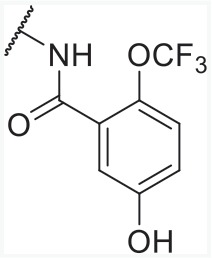	H	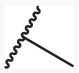	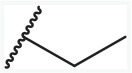	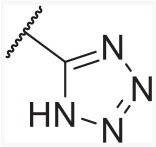	-0.724	-1.525
**8**	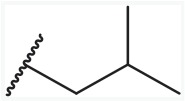	H	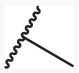	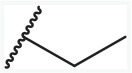	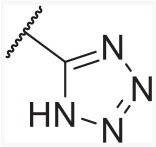	-0.418	-2.326
**19**	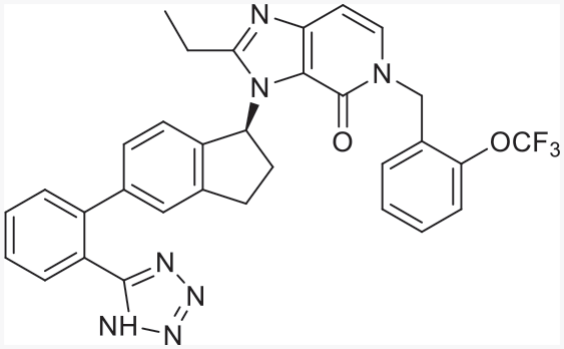					-1.466	-1.304

## MATERIALS AND METHODS

### Data set

A set of imidazo-\ pyridines with dual activities towards AT1 antagonism (IC_50_: 0.49∼94.1 nM) and PPARγ partial activation (EC_50_: 20∼3640 nM) (Table 1) were collected from the published literatures [17, 29]. In the study, compounds with highly structural difference and no explicit activity values were excluded and not applied into the modeling. For modeling convenience, the eventual 26 molecules including telmisartan, 10 imidazo[4,5-b]pyridines and 15 imidazo[4,5-c] pyridin-4-one derivatives were selected and renumbered. The values of AT1 antagonistic activity and PPARγ partial activation were changed into the corresponding pIC_50_ (-log IC_50_) and pEC_50_ (-log EC_50_) respectively as dependent variables in the study. These molecules were divided into a training set and a test set randomly with a certain proportion (4:1) aiming to include the structural and biological diversity [31, 32]. The former set was used to conduct the predictive CoMFA model while the latter was to validate and evaluate the predictability [33]. In this work, training sets of 21 molecules and test sets of 5 molecules respectively for CoMFA models (AT1 and PPARγ) were divided.

### Construction and optimization of 3D structures

Before modeling, the planar structures of these imidazo-\ pyridine derivatives were first sketched utilizing ChemBioDraw software. Through the transformative function of “3D Geometry (Concord)” protocol implemented in SYBYL-X 2.1 software, we received the 3D structures of 26 derivatives. Afterwards, the optimized 3D conformations were obtained by “minimize” command using Powell method and Tripos force field. With the Gasteiger-Huckel Charges computation method, the process was initially performed in a simplex way and the termination value was set to gradient 0.005kcal/mol·Å and max iterations to 1000 [34]. Other settings in this module were default.

### Preparation of receptors and small ligands

Prior to docking, all preparations of receptors (AT1 and PPARγ) and ligands were done via the corresponding panels implemented in Maestro v10.2 (Schrödinger, LLC, New York, 2015) [35]. The proteins for docking analysis were downloaded from RCSB Protein Data Bank (http://www.rcsb.org/pdb/home/home.do, PDB ID: 4ZUD for AT1, 3R8A for PPARγ) [17, 36]. All these compounds were docked into the receptor's active binding cavity.

As to the optimization of receptors (AT1 and PPARγ), we utilized “Protein Preparation Wizard (PPW)” protocol to assign bond orders, add hydrogens, create zero-order bonds to metals, create disulfide bonds, cap termini and delete waters [37]. For the sake of optimizing the -OHs orientation and regulating the state of some amino acids, the H-bond optimization was proceeded accordingly. Energy minimization was monitored with the root mean square deviation (RMSD) set to 0.5 and the force field environment to Optimize Potentials for Liquid Simulations 2005 (OPLS_2005) [38]. Then all molecules were assigned to “LiPrep” module (Schrödinger, LLC, New York, 2015) for preparation [39, 40]. The pH condition for ionization generation was set to 7.0 +/− 2.0 while the force field to be default OPLS_2005 as receptor preparation to avoid bonds crash.

### Molecular docking study

Once the cubic grid box (10×10×10 Å for AT1 and 12×12×12 Å PPARγ) around the active site residues generated, the prepared molecules and proteins were submitted to Glide docking panel (Schrödinger, LLC, New York, 2015) for a perfect docking analysis [41]. According to the subsequent docking results, we were able to analyze the binding stability of a ligand-protein complex and the matching degree in the binding surface.

### Molecular alignment

**Figure 11:**
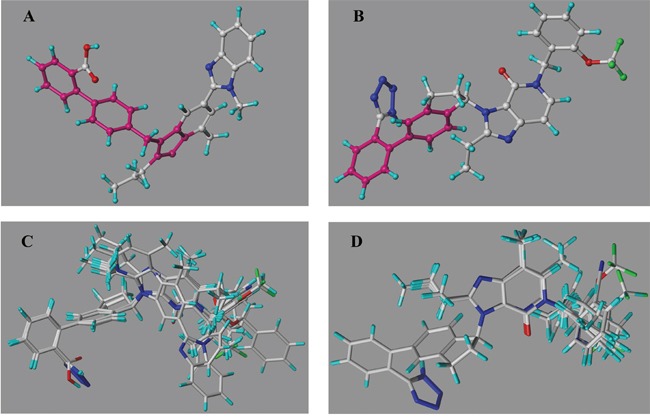
**(A)** The common substructure (magenta section) on the template of telmsartan (AT1 model) for superimposition **(B)** The common substructure (magenta section) on the template of 19 (PPARγ model) for superimposition. **(C)** The molecular alignment of the training set on AT1 model. **(D)** The molecular alignment of the training set on PPARγ model.

A perfect database alignment is crucial for deriving a reliable CoMFA model and improving the predictability [31, 42]. “Align Database” panel in SYBYL-X 2.1 was adopted to superimpose the compounds set to the template molecule when fixed the common substructure (Figure 11). Considering the configurations in imidazo-\ pyridine derivatives, the common moieties of these two groups in this study were determined as indicated in Figure 11A and 11B. The reference structures for superimposition were the most active telmisartan (AT1) and 19 (PPARγ), respectively.

### CoMFA models

After database alignment, the activity values of these derivatives were imported to calculate the CoMFA descriptors. CoMFA analysis is a method involving the shapes of molecules. It operated on energy values at points in space surrounding the molecules. Efforts to construct better model, 30.0 kcal/mol cutoff values for steric and electrostatic fields were assigned in creating Tripos Standard field. The energy calculations of steric (Lennard-Jones 6-12 potential) and electrostatic (Coulombic potential) fields were done automatically by the default setting, using a probe atom with van der Waals (vdW) properties of c.3 (sp^3^ carbon as steric probe) and a charge of +1 (as electrostatic probe) [43]. Every molecule was set in 2Å spacing in all directions. The distance was selected as the dielectric value to control the form of the Coulombic electrostatic energy calculation [44].

When the parameters of the model calculated, Partial Least Squares (PLS) method was adopted to derive linear relationships between the bioactivity values and the CoMFA descriptors [37, 45]. PLS analysis is generally performed in two stages. The first stage is with Leave-One-Out (LOO) method to determine how rich or complex a model is appropriate for the data values or how many components to use. LOO method means one molecule was removed from the data set and the activity was predicted by the rest [46]. Along with the optimum number of components (ONC) and no validation method, the second stage was to establish the single model that best represents the data. The cross-validated correlation coefficient q^2^ produced from the internal test determined the goodness-of-fit of the model [47]. This value was calculated using the following equation:

$${\text{q}}^{2}=1-\frac{{\sum \left(Y_{pred}-Y_{act}\right)}^{2}}{{\sum \left(Y_{act}-Y_{mean}\right)}^{2}}$$

Where *Y_pred_* and *Y_act_* refer to the predicted and actual activities of each molecule towards single target, respectively; *Y_mean_* is the mean activities of whole training set.

Other statistical outcomes yielding from stage two to evaluate the fitting capability, robustness and stability of the model were standard error of estimate (SEE), the conventional correlation coefficient (r^2^), Fisher Test (F) value and fields (steric and electrostatic) contributions. If q^2^ value is below 0.5 or r^2^ no greater than 0.6, the model is indicated to be relatively poor [48]. Additionally, the closer the SEE value is to 0 and the larger value to F, the higher predictivity the model will be [32].

Once the CoMFA model of the training set constructed completely, the test set not involved in the modeling was used to test the external predictivity and if the model is appropriate and robust through r_pred_^2^ [49]. Based on the StDev*Coefficient (the standard deviation and the coefficient) contour maps, the specific impact of steric or electrostatic field contribution and distribution on potential activity would be viewed clearly [50]. All the calculations were operated in CoMFA protocol of SYBYL-X 2.1 software package.

## CONCLUSIONS

Imidazo[4,5-b]pyridines and imidazo[4,5-c] pyridin-4-one derivatives modified from telmisartan have been identified with dual AT1 antagonistic and PPARγ partial agonistic activity. In this work, the docking simulation and 3D-QSAR analysis were performed to study the SAR as well as the binding mechanism of imidazo-\pyridines with AT1 and PPARγ pockets. Docking results demonstrated the interaction modes and the matching degree with the binding surface. Specifically, the binding modes between imidazo-\pyridines and PPARγ active cavity were validated to be totally opposite from that of typical activators. From the best CoMFA models, high values for q^2^, r^2^ and r_pred_^2^ (q^2^>0.5, r^2^>0.8, r_pred_^2^>0.6) indicated satisfactory internal and external predictivity. Additionally, we concluded: (1) Increasing the R_1_ substituent properly will be beneficial to enhance PPARγ partial activity and maintain AT1R antagonistic activity; (2) The electronagative groups like trifluoromethoxy in C-2 of part R_1_ caused the dual activities to increase and compounds with 2-substituted electropositive groups tended to be more active than that of other positions; (3) R_2_ substitution was improper for enhancing the activities towards AT1R antagonism and PPARγ partial activation; (4) ethyl or propyl in R_4_ was appropriate for dual activities, larger substituents were unworkable; (5) Tetrazole ring or carboxylic acid in R_5_ was responsible for better dual activities. The successful molecules design based on the contour maps of steric and electrostatic fields illustrated that the constructed CoMFA models were highly stable and practicable to acquire novel, potential dual AT1/PPARγ agents. Docking results were roughly coincident with the CoMFA contour maps. CoMFA models of both targets integrated with the docking analysis will be of great benefit in the optimization of potential dual AT1 antagonists and PPARγ partial agonists and in the identification of novel leads.

## Abbreviations

AT1R: angiotensin II type 1 receptor
PPARγ: peroxisome proliferator-activated receptor γ
QSAR: Quantitative structure-activity relationships
T2DM: Type 2 diabetes mellitus
GPCR: G protein-coupled receptor
Ang II: angiotensin II
ARBs: AT1 receptor blockers
SAR: structure-activity relationship
CoMFA: Comparative Molecular Field Analysis
PDB: Protein Data Bank
PPW: Protein Preparation Wizard
RMSD: root mean square deviation
OPLS_2005: Optimize Potentials for Liquid Simulations 2005
PLS: Partial Least Squares
LOO: Leave-One-Out
ONC: optimum number of components
SEE: standard error of estimate
SP: standard-precision
StDev*Coeff: the standard deviation and the coefficient.
